# Debulking Hepatectomy for Colorectal Liver Metastasis Conveys Survival Benefit

**DOI:** 10.3390/cancers16091730

**Published:** 2024-04-29

**Authors:** Jennifer A. Kalil, Lucyna Krzywon, Oran Zlotnik, Hugo Perrier, Stephanie K. Petrillo, Prosanto Chaudhury, Erik Schadde, Peter Metrakos

**Affiliations:** 1 Department of Surgery, Royal Victoria Hospital—McGill University Health Center, 1001 Blvd Décarie, Montréal, QC H4A 3J1, Canada; jennifer.kalil@mail.mcgill.ca (J.A.K.); lucyna.krzywon@mail.mcgill.ca (L.K.); oran.zlotnik@mail.mcgill.ca (O.Z.); hugo.perrier@mail.mcgill.ca (H.P.); prosanto.chaudhury@mcgill.ca (P.C.); 2 Cancer Research Program, Research Institute—McGill University Health Center, 1001 Blvd Décarie, Montréal, QC H4A 3J1, Canada; stephanie.petrillo@muhc.mcgill.ca; 3 Hepatobiliary and Pancreatic Surgery, Surgical Center in Zurich, Surgery Center St. Anna in Lucerne, Beausite Hospital in Berne, Hirslanden Corporation, Witteliker Str. 40, 8032 Zurich, Switzerland; erik.schadde@icloud.com; 4 Department of Surgery, Rush University Medical Center, 653 W Congress Pkwy 12, Chicago, IL 60612, USA

**Keywords:** colorectal liver metastases, debulking hepatectomy, chemotherapy, liver resection, median survival

## Abstract

**Simple Summary:**

Despite advances in surgical techniques and systemic therapy, some patients with multifocal, bilobar colorectal liver metastases remain unresectable. The surgical debulking of liver tumor burden may increase median survival in combination with chemotherapy compared to chemotherapy alone. This study retrospectively evaluated the efficacy of surgical debulking alongside chemotherapy versus chemotherapy alone and found those who underwent debulking surgery showed improved survival compared to those who did not. These findings advocate for further investigation through a randomized trial to evaluate intentional debulking as a potential treatment strategy for unresectable colorectal cancer liver metastases.

**Abstract:**

(1) Background: Despite advances in surgical technique and systemic chemotherapy, some patients with multifocal, bilobar colorectal liver metastases (CRLM) remain unresectable. These patients may benefit from surgical debulking of liver tumors in combination with chemotherapy compared to chemotherapy alone. (2) Methods: A retrospective study including patients evaluated for curative intent resection of CRLM was performed. Patients were divided into three groups: those who underwent liver resection with recurrence within 6 months (subtotal debulked, SD), those who had the first stage only of a two-stage hepatectomy (partially debulked, PD), and those never debulked (ND). Kaplan–Meier survival curves and log-rank test were performed to assess the median survival of each group. (3) Results: 174 patients underwent liver resection, and 34 patients recurred within 6 months. Of the patients planned for two-stage hepatectomy, 35 underwent the first stage only. Thirty-two patients were never resected. Median survival of the SD, PD, and ND groups was 31 months, 31 months, and 19.5 months, respectively (*p* = 0.012); (4) Conclusions: Patients who underwent a debulking of CRLM demonstrated a survival benefit compared to patients who did not undergo any surgical resection. This study provides support for the evaluation of intentional debulking versus palliative chemotherapy alone in a randomized trial.

## 1. Introduction

Colorectal cancer remains the third most common cause of cancer and cancer-related mortality worldwide [1]. Approximately half of the patients diagnosed with colorectal cancer develop colorectal liver metastasis (CRLM) over the course of their disease, and surgical resection remains the only treatment option capable of providing long-term survival and sometimes cure [2]. Up until the 1990s, liver resection for CRLM was not recommended due to both the high morbidity and mortality of hepatectomy in that era and the uncertainty as to whether the surgical removal of metastases would offer patients a survival benefit [3,4]. 

### 1.1. Extending Oncological Resectability

With advances in chemotherapy and surgical techniques, resection became more common, and early studies demonstrated a significant survival benefit after liver resection for CRLM with an acceptable perioperative mortality. This encouraged surgeons to expand the criteria for oncological resectability for CRLM over the next two decades [5,6,7]. As the criteria for resectability continued to change, systemic chemotherapy was also evolving. With more effective chemotherapeutic regimens, patients who were expected to have close or even positive margins by the proximity of the tumors to vascular structures were being considered for operative therapy. In patients with an optimal response to preoperative chemotherapy seen on imaging, no difference was observed in 3-year overall survival (OS) between patients who underwent R0 vs. R1 resection, despite the fact that only 8% of patients with R1 liver resections were cured [8,9].

### 1.2. Extended Criteria for Resection of CRLM

Generally, most resections for CRLM are performed with curative intent; however, there is evidence that in some subgroups of patients—like those with extrahepatic disease and those with extensive liver tumor load, the cure is very rare. Despite this, surgeons and oncologists are recommending combinations of the resection and ablation of CRLM, knowing that survival may be prolonged as long as the interventions do not incur major complications [10,11].

The potential next step in the evolution of management for CRLM is compelling—do patients benefit from a non-curative intent resection, or “debulking”, by decreasing overall liver tumor burden? Is there a role for liver tumor volume reduction as an extended criterion to improve median survival?

The aim of this study is to explore the survival benefit of such “debulking resections” for unresectable CRLM. Since good clinical practice does not allow for liver resections to be routinely performed for unresectable CRLM or in cases where large tumor volume would be left in situ outside of a prospective research protocol, debulking can only be studied retrospectively by exploring the outcome of patients undergoing failed TSH or in patients with early liver recurrence after curative intent resection. This single-center study compares the survival in these groups that resemble a debulking approach to a group that did not undergo liver resection at all.

## 2. Materials and Methods

### 2.1. Patient Selection

The institutional review board at McGill University approved this retrospective study, which included all patients who had consented to participate in the McGill University Liver Disease Biobank research program between 2012 and 2020. Patients over the age of 18 with CRLM who were evaluated for curative intent resection were included in the analysis.

All patients were evaluated by a multidisciplinary tumor board (MDT) consisting of hepatobiliary surgeons, medical oncologists, radiologists, and pathologists. For patients that were not “upfront resectable” (i.e., requiring downsizing neoadjuvant chemotherapy, TSH, or regenerative liver surgery), the standard management included first-line systemic chemotherapy with routine evaluation by the oncologist and surgeon assessing radiological response to determine the optimal time for surgery. Patients with similar performance status, extent of hepatic metastatic disease burden, and either response to or no progression of disease while receiving chemotherapy were included in the study. Patients who were considered never resectable, those with carcinomatosis, and patients whose disease progressed while in chemotherapy were excluded from the study. Additionally, patients with missing data were also excluded. 

### 2.2. Study Design and Group Definitions

**Figure 1:**
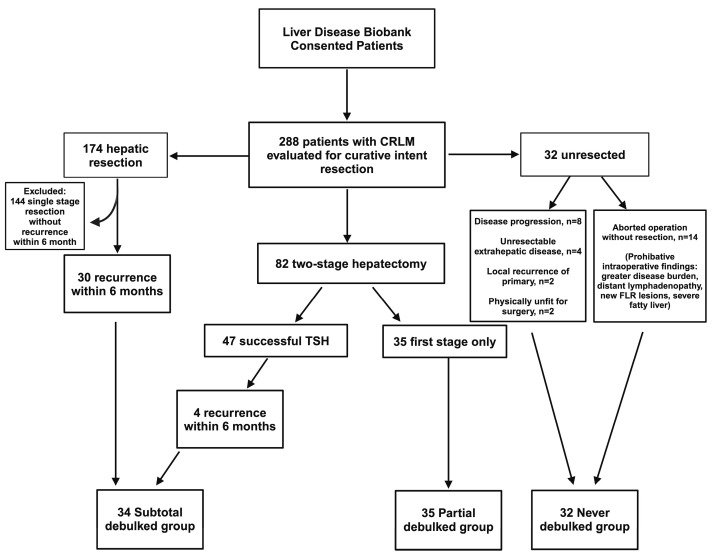
Allocation of patients into groups. Patients who underwent single-stage partial hepatectomy or successful completion of TSH with recurrence within 6 months were allocated into the subtotal debulked group. Patients who underwent first stage only of TSH approach were allocated to the partial debulked group. Patients who were never resected were allocated to the never resected group. TSH, two-stage hepatectomy; CRLM, colorectal liver metastasis.

Patients assessed for curative resection were retrospectively categorized into three groups, as outlined in Figure 1. Those failing TSH (successful completion of first stage but did not undergo or complete second stage) and those with recurrence within 6 months of curative intent resection were best surrogates of patients undergoing a debulking operation, as described in previous studies [12]. The group of patients with failed TSH were called the “partial debulked” group, and those with early recurrence, within 6 months after curative intent resection, were called the “subtotal debulked” group. The “never debulked” group was comprised of patients with aborted resections or diseases unresponsive to chemotherapy. All groups adhered to identical selection criteria to ensure methodological consistency and minimize confounding factors. This ensured homogeneity across all groups to enhance the comparability of the study outcomes. 

### 2.3. Surgical Strategy

Our institution’s approach to managing complex cases, particularly with high liver tumor burden, involves a multidisciplinary approach integrating surgical intervention with various liver-directed therapies, including hepatic artery infusional methods, transarterial embolization techniques, and local ablative therapies, when it is necessary to achieve curative outcome. Interventional procedures were applied as indicated for all patients across the three groups. 

All operations were performed through an open approach and completed by three different surgeons. Central venous access was obtained, and intraoperative ultrasound was performed in every case. During parenchymal transection, a central venous pressure of less than 5 mmHg was maintained. Resections were performed utilizing an anatomic/segmental approach, with parenchymal division accomplished through water-jet dissection. 

Postoperative morbidity was categorized according to the Clavien-Dindo classification of surgical complications [13]. Post-hepatectomy liver dysfunction was defined according to the International Study Group of Liver Surgery (ISGLS) consensus definition and severity grading of post-hepatectomy liver failure (PHLF) [14]. Patient demographics and clinical characteristics between the groups were analyzed for prognostic factors. Overall survival was the primary endpoint of the study, which was defined from the date of liver metastasis diagnosis to the date of death/last clinic visit. The patients that underwent resection were seen in clinic approximately two weeks postoperatively and every three months thereafter.

### 2.4. Statistical Analysis

Continuous variables with normal distribution are reported by mean ± standard deviation (SD) and compared with *t*-test or ANOVA. Continuous variables with abnormal distribution are reported as median (range) and compared with Mann–Whitney U test and Kruskal–Wallis tests. Comparisons of categorical variables between groups were compared with Chi-square test. Cox proportional hazard models were used for univariable and multivariable analyses to identify factors associated with survival. Survival analyses were estimated using Kaplan–Meier prediction, and differences between groups were tested using log-rank test. Statistical analyses were run in GraphPad Prism (version 9.4.1 GraphPad Software LLC, Boston, MA, USA) and R Statistical Software (v4.2.1, R Core Team 2022, Vienna, Austria).

## 3. Results

### 3.1. Patient Group Allocation

During the study period, 288 patients with CRLM evaluated for curative intent resection were identified from the liver disease Biobank (Figure 1). Of these, 174 patients underwent a single-stage partial hepatectomy, of which 30 patients developed early recurrence (defined as liver recurrence within 6 months of hepatic resection). TSH was attempted in 82 patients. TSH was successfully completed in 47 patients (57%), with 4 patients developing early recurrence, while 35 patients (43%) underwent the first stage only. Reasons for failed TSH included tumor progression, new lesions in the FLR, insufficient growth of the FLR, or patient deconditioning. Thirty-two patients did not undergo any liver resection due to the progression of disease on best chemotherapy (n = 8), unresectable extrahepatic disease (n = 4), local recurrence of primary (n = 2), or deconditioning due to chemotherapy (n = 2). There were 14 patients who had an aborted liver resection after exploration revealed prohibitive intraoperative findings (i.e., greater than anticipated disease burden, distant lymphadenopathy, new lesions in the FLR, or severe fatty infiltration of the liver).

### 3.2. Patient Demographics and Characteristics

The patient demographics and characteristics are summarized in Table 1 and were similar across the three groups. There was no difference in baseline CEA, the location of the primary tumor, the location of liver lesions, or the size of the largest liver lesion between the groups. More than 70% of patients in each group presented with a synchronous disease. The subtotal debulked group had significantly fewer liver lesions compared to the partial and never debulked groups (three lesions (range: 1–6), vs. five lesions (range: 1–9), vs. four lesions (range: 1–16), respectively; *p* < 0.001), and there was no difference in number of liver lesions between the partial and never debulked groups (*p* = 0.99). Additionally, more patients in the subtotal debulked group had unilobar liver lesions (*p* < 0.001, 95% CI 2.8–26.2), while there was no difference in the location of liver lesions between the partial and never debulked groups (*p* = 0.35, 95% CI 0.16–1.86). Of the 35 patients in the partial debulked group, 18 patients (51%) had <50% tumor volume resected in the first stage of the failed TSH. 

### 3.3. Perioperative Course and Postoperative Outcomes

In the subtotal debulked group, patients experienced recurrence within 6 months as per the group’s predefined criteria. Of the 34 patients in this group, 24 patients presented with synchronous disease, with 7 patients undergoing a liver-first approach, 2 patients undergoing simultaneous resection of primary and liver metastases, and 9 patients undergoing colon resection first. Each of the 10 patients who presented with metachronous disease had previously undergone a resection of their primary colon cancer. Additionally, 79% of patients in this group received neoadjuvant chemotherapy prior to liver resection (median 6 cycles, range 2–12 cycles), and 80% received adjuvant chemotherapy (median 5 cycles, range 2–6 cycles). 

In the partial debulked group, 23 of 35 patients had their primary tumor resected, among whom 3 presented with metachronous disease. Of the 32 patients with a synchronous disease, the liver-first approach was performed in 11 patients, 4 patients underwent simultaneous resection of the colon primary and liver metastases, and the colon-first approach was performed in 17 patients. Neoadjuvant chemotherapy was given to 91% of patients prior to liver resection (median 6 cycles, range 3–16), and 87% received adjuvant chemotherapy (median 6 cycles, range 3–22 cycles).

The median length of follow-up was 20 months (range 2–75 months). Surgical complications, according to the Clavien-Dindo classification, are listed in Table 1 [13]. Twelve patients (20%) had grade I-II complications, while three patients (5%) had complications classified as grade III-IV. There were no mortalities within 30 days of surgery. Transient Grade A and B PHLF measured on postoperative day 5 was identified in two and one patient(s), respectively (Table S1).

### 3.4. Prognostic Factors and Overall Survival

**Figure 2:**
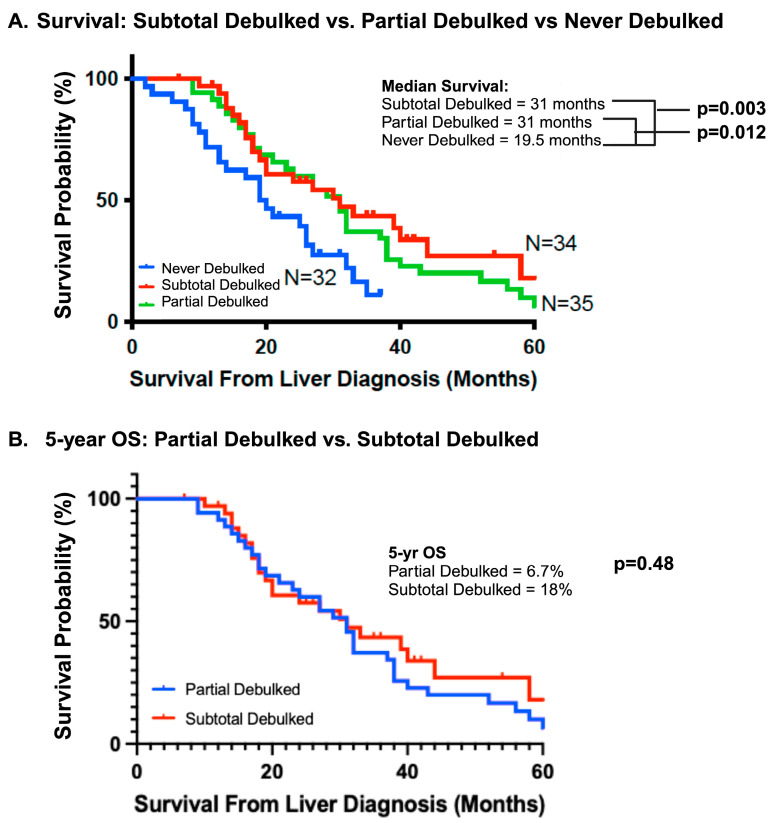
Survival outcomes. (**A**) The median survival of the subtotal debulked, partial debulked, and never debulked groups. (**B**) Five-year overall survival outcomes between the partial debulked and subtotal debulked groups. OS, overall survival.

**Figure 3:**
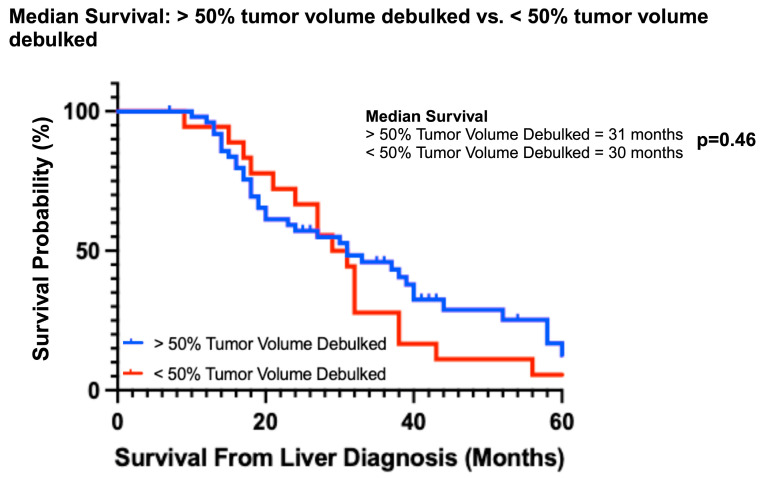
Median survival based on percent tumor volume debulked (> or <50%).

Overall survival was better for both the partial and subtotal debulked groups compared to the never debulked group (Figure 2A). The median OS of the never debulked group was 19.5 months (2–37 months), and there were no survivors at 5 years. The median OS in both partial and subtotal debulked groups was 31 months (PD: 12–86 months; SD: 10–64 months). The 5-year OS was 6.7% and 18% in the partial and subtotal debulked groups, respectively (*p* = 0.48) (Figure 2B). The univariable analysis demonstrated a survival advantage for patients in both the partial (hazard ratio 0.48, 95% CI 0.27–0.85; *p* < 0.01) and subtotal debulked groups (HR 0.42, 95% CI 0.23–0.77; *p* < 0.01) compared to the never debulked group (Table 2). When the partial debulked group was further stratified into >50% overall tumor burden resected and <50% overall tumor burden resected, there was no difference in the median survival (31 months and 30 months, respectively; *p* = 0.46) between these two subgroups (HR 0.57, 95% CI 0.83–3.78; *p* = 0.14) (Figure 3). 

Univariable and multivariable analyses were conducted to evaluate possible clinicopathologic risk factors influencing survival, as detailed in Table 3. Given the absence of significant associations revealed by the univariable analysis between the clinical characteristics and survival outcomes, Fong clinical risk scores were calculated to leverage established parameters associated with poor prognosis [15]. The median score in each group was 3, with a mean ± SD of 2.69 ± 1.1, 3.28 ± 0.8, and 2.96 ± 1.3 in the SD, PD, and ND groups, respectively (*p* = 0.06). A multivariable analysis was conducted to explore potential biases influencing retrospective group allocation. This involved a comparison of each clinicopathologic factor against the others to ascertain if any interdependencies existed that could affect survival outcomes. Again, none of the clinicopathologic variables in the analysis demonstrated an impact on survival. 

The analysis was then repeated, incorporating group allocation into the multivariable model to examine the potential effects of the same clinicopathologic factors on survival after accounting for group differences. This analysis showed HR 0.49 (95% CI 0.23–1.04, *p* = 0.06) in the partial debulked and HR 0.43 (95% CI 0.17–1.05, *p* = 0.06) in the subtotal debulked group, while repeat analysis with the partial and subtotal debulked group as a single cohort demonstrated a statistically significant survival benefit (HR 0.47, 95% CI 0.23–0.96, *p* = 0.03) (Table 3). 

## 4. Discussion

This study challenges the concept that complete resection is necessary to provide a survival benefit for patients with CRLM and demonstrates that partial resection, or debulking, of metastatic liver disease may provide a significant improvement in survival. A multivariable analysis to correct for confounders did not change this result significantly. Stratification by the amount of tumor volume resected did not significantly impact survival and demonstrated that any tumor volume resection results in better survival compared to no resection at all. The study, therefore, lends support to the concept of debulking hepatectomy over no resection.

Although prospective studies investigating the efficacy of debulking for CRLM are lacking, the existing literature does indicate that incomplete resection of the disease confers a survival benefit. The CLOCC trial (EORTC 40004 CLOCC, No. NCT0043004) compared OS in patients with unresectable CRLM who were randomized to receive systemic treatment alone or systemic treatment plus aggressive local treatment with curative intent by radiofrequency ablation with or without resection [16]. Patients in the combined modality arm had a statistically significantly longer OS compared to the patients receiving systemic treatment alone (45.6 months vs. 40.5 months, *p* = 0.01) [16]. The median progression free survival was almost twice as long as in the combined modality group (16.8 months) compared to the systemic treatment group (9.9 months) [16]. The results of this study demonstrate how a combination of resection and ablation of unresectable CRLM may improve overall survival and is the only randomized trial in the literature to support the concept of resection over chemotherapy alone in the field of CRLM.

The resection of CLRM in the presence of extrahepatic disease improves survival over palliative chemotherapy [17]. Factors impacting survival include the site of EHD (pulmonary or extrapulmonary) and its resectability. Patients with resectable pulmonary metastases have a longer median OS than those with resectable lymph node metastases (portal, mesenteric, or retroperitoneal) [18,19]. Furthermore, patients with both CRLM and colorectal lung metastases experience improved survival after liver resection compared to those undergoing only chemotherapy [17]. Patients with resection of both liver and lung metastases had the best OS compared to those with resection of CRLM only. Interestingly, resection of only the liver metastases while leaving the lung metastases was still better than the chemotherapy-only group (5-year OS 56.9% vs. 13.1% vs. 1.6%, respectively; *p* < 0.01) [17]. Similarly, the 5-year OS after CRLM resection in a cohort of patients from LiverMetSurvey without resection of lung metastases was 14.3% [20]. Despite inferior survival outcomes compared to patients undergoing curative-intent resection plus chemotherapy, a statistically significant survival benefit persists in this group of incompletely resected (i.e., only the liver metastases, not the lung metastases) patients. 

Allard et al. investigated the survival outcomes in patients with 1–3, 4–9, or ≥10 liver metastases after liver resection [21]. This study demonstrated that patients with ≥10 CRLM and at least one favorable prognostic factor achieved a 5-year OS of 21% and median survival of 34 months after undergoing a “macroscopically complete resection” [21]. Conversely, Viganò et al. highlight the significant challenge and poor prognosis of very early recurrence within three months post-hepatectomy [22]. Despite this, the study shows that an aggressive re-intervention rate could potentially salvage long-term outcomes in very select cases [22]. Similarly, additional investigations have explored the efficacy of combining radiofrequency ablation with hepatectomy for initially unresectable disease, demonstrating comparable OS and DFS outcomes to those achieved with TSH or hepatectomy alone [23,24]. The findings of these studies collectively underscore the potential of a comprehensive and aggressive oncosurgical approach to achieve prolonged survival outcomes effectively. An interdisciplinary approach with robust follow-up and readiness to re-engage with curative intent strategies are critical in managing recurrence and improving chances of survival. 

Liver tumor debulking may have potential benefits, some of which have been observed in small retrospective series or predictive analyses [25,26]. Currently, there are three working hypotheses to explain the effectiveness of debulking CLRM. 

First, it can be speculated that a reduction of extensive CRLM may prevent future liver dysfunction. Debulking of liver metastases prevents the liver from developing progressive metabolic dysfunction and death from terminal cancer [27]. It is known that patients with liver metastases die of progressive jaundice, leading to anorexia, weight loss, generalized disability, and death. In a study of 476 patients with stage IV colorectal cancer, Stewart et al. found liver involvement in 83% of cases at death, with the liver being the primary site of metastatic disease in 49% [27]. Additionally, patients who underwent hepatectomy had a lower liver disease mortality rate than those with unresected CRLM (32% vs. 71%; *p* < 0.0001) [27].

Second, reducing tumor burden may lessen the immunosuppressive effects of the liver tumor mass, enhancing the host immune response against tumor antigens [28]. While mechanistic data supporting this hypothesis are scarce, growing evidence suggests liver metastases convey a tumor-specific immune suppression. Preclinical models demonstrate liver metastasis hinders immunotherapy response by depleting both systemic tumor-specific CD8+ T cells and hepatic-derived macrophage-induced antigen-specific T cell apoptosis [29]. Liver-directed radiotherapy may also reprogram the tumor microenvironment from being immune suppressive to stimulating antitumor immunity [29,30]. It is, therefore, conceivable that surgical debulking may offer the same attenuation in the hepatic immune response. Other studies demonstrate that checkpoint inhibitors work better in patients without liver metastases [31,32]. With this in mind, it is reasonable to hypothesize that a similar transition to an onco-immunostimulatory or a less onco-immunosuppressive environment occurs after tumor debulking surgery. 

Third, debulking CRLM may reduce the genetic heterogeneity of the tumor and, thereby, chemotherapy resistance [33]. Spatial transcriptomics characterizes gene expression in primary tumors, metastatic liver lesions, and intralesional tumor cells [34]. Genetic alterations in tumor clones and intratumoral heterogeneity may confer acquired therapy resistance [33,34]. It may be postulated that decreasing tumor volume could decrease heterogeneity and enhance treatment response. However, comprehensive mutational analysis for each of the metastatic tumors in patients after complete resection and debulking hepatectomy is lacking to validate this hypothesis.

A study by Brouquet et al. underscores the complexity of managing patients with CRLM, particularly in the context of TSH [35]. Of the patients that completed TSH, 62% developed recurrence, with a 1-year disease-free survival of only 39% [35]. The subtotal debulked group presumes recurrence within 6 months results from residual disease post-surgery. The rationale behind using early recurrence as a surrogate for subtotal debulking stems from recognizing it may offer a pragmatic strategy to mitigate tumor burden with the aim of achieving maximal disease control and potentially prolonging survival, even in the face of early recurrence. 

The partial debulked group is the best representation of debulking surgery because these patients underwent resection while knowingly leaving some tumor in situ as part of a planned two-stage approach. Brouquet et al. found patients undergoing only the first stage of a TSH derived no survival benefit and had worse survival compared to chemotherapy alone [35]. The discrepancy in findings may stem from several factors. First, the present study included a larger cohort of patients who did not complete TSH, nearly doubling the sample size (35 patients vs. 18 patients), potentially accounting for significant differences in survival. Second, it is not clear if additional therapeutic modalities were offered to TSH failure patients. At our institution, a multidisciplinary approach is the cornerstone of managing complex cases of initially unresectable or extensive liver tumor burden. This comprehensive strategy integrates surgical intervention with liver-directed therapies through hepatic artery infusional methods, transarterial embolization techniques, and local ablative therapies, all aimed at achieving a curative outcome. Consequently, these strategies are frequently utilized following unsuccessful attempts of surgery with curative intent and likely contribute to the survival benefit observed in debulked patients. 

The current study has several limitations. The limited sample size increases the susceptibility to a type-II error. In instances where significant associations were not observed for established poor prognostic factors, Fong scores were additionally computed as a surrogate. Moreover, the retrospective assignment of patients to groups based on approximate tumor volume resected could subject the findings to selection bias. To mitigate this, stringent inclusion and selection criteria were used to ensure comparability between the groups, but due to the retrospective design, the risk of bias cannot be completely excluded. Therefore, the findings of this study cannot be considered conclusive but rather a stimulus for further investigation and challenging the current paradigms in the field. It is important to understand the limitations and potential biases within each group while simultaneously recognizing there are no guidelines for debulking CRLM. Defining these surrogate groups remains the best option for survival analysis, given the current practice standards. 

The data thus far support a call for a prospective randomization of “never debulked” patients into a debulking and chemotherapy-only group. The only randomized data in patients with unresectable CRLM are from the CLOCC trial, and in that study, the therapeutic group underwent complete eradication of the tumor with curative intent with the help of ablation [16]. Now may be the only time to determine if survival can be improved with debulking compared to chemotherapy alone in a prospective fashion, although such a trial would pose unique challenges. Without ample data to support debulking, there would be both recruitment challenges and ethical concerns regarding group allocation. However, the key to a prospective study would be a strategy for evaluating the trade-off of undesirable consequences from the surgical treatment, such as pain, complications, and mortality, and should include the assessment of both the post-surgical morbidity and the complications and negative side effects of chemotherapy treatment in the same descriptive currency. This will allow a direct comparison of the negative side effects of both therapies against each other. After all, palliative chemotherapy also impacts quality of life, and since survival may not be the ultimate endpoint of modern cancer treatment, quality-of-life-adjusted survival may be the ideal endpoint for such a study. 

## 5. Conclusions

While intentional surgical debulking is not an accepted practice based on published data and guidelines, this study contributes to our understanding by offering a detailed retrospective analysis of prolonged survival outcomes associated with surgical debulking of CRLM. This adds a novel perspective to the field, which has not been extensively explored previously. However, due to the retrospective nature of this study, it remains unclear at what cost to quality of life such prolongation of survival is achieved. These retrospective data require the evaluation in a randomized study with the endpoint of quality of life-adjusted survival for patients with unresectable liver metastases undergoing either intentional debulking versus palliative chemotherapy alone. 

## Figures and Tables

**Table 1:** Patient demographic and clinical characteristics by group.

	Subtotal Debulked (n = 34)	Partial Debulked (n = 35)	Never Debulked (n = 32)	*p* Value
Age (years)	61 (+/−10.4)	58.2 (+/−10.1)	59.2 (+/−13.1)	0.58
Male/female	20:14	26:9	12:20	0.14
BMI (kg/m^2^)	26.9 (+/−4.9)	26.7 (+/−3.63)	25.9 (+/−4.8)	0.74
CEA (ng/mL)	5.25 (1.1–3610)	26.5 (1.5–9493)	12.4 (1.1–12,355)	0.10
Number of liver lesions	3 (1–6)	5 (1–9)	4 (1–16)	<0.001
Diameter largest liver lesion (cm)	3.4 (+/−3.29)	3.13 (+/−2.89)	2.9 (+/−3.38)	0.93
Fong score [15]	3 (1–5)	3 (1–5)	3 (0–5)	0.06
Location of liver lesions				<0.001
Unilobar	21	4	7	
Bilobar	14	31	23	
Location of colon primary				0.25
Right	11	12	13	
Left	7	9	10	
Rectum	15	11	5	
Synchronous presentation	24 (70.5%)	32 (91%)	27 (84%)	0.08
Perioperative chemotherapy	32 (94%)	34 (97%)	31 (97%)	0.84
Histologic growth pattern of CRLM				0.62
Desmoplastic	11	20	N/A	
Replacement	23	14	N/A	
Clavien-Dindo Classification [13],				
n (%)				
0	25 (75.8%)	20 (74%)		
I	3 (9%)	1 (3.7%)		
II	4 (12%)	4 (14.8%)		
III	1 (3%)	1 (3.7%)		
IV	0	1 (3.7%)		
V	0	0		

Data presented as mean (+/− standard deviation) or median (range). BMI, body mass index; CEA, carcinoembryonic antigen; CRLM, colorectal liver metastasis; N/A, not applicable.

**Table 2:** Impact of surgical debulking on median and overall survival.

Group	Median Survival (Months)	5-Year OS	HR	95% CI	*p* Value
Never debulked	19.5	0%			Reference
Partial debulked	31	6.7%	0.48	0.27–0.85	<0.01
Subtotal debulked	31	18% *	0.42	0.23–0.77	<0.01

HR, hazard ratio; OS, overall survival. * Comparison of 5-year OS between the two debulking groups was not significant (*p* = 0.48).

**Table 3:** Univariable and multivariable analysis of clinicopathologic factors on survival before and after group stratification.

	Univariable		Multivariable *			
			Before Group Stratification		After Group Stratification	
Variable	HR (%95 CI)	*p*	HR (%95 CI)	*p*	HR (%95 CI)	*p*
Age (years)	0.76 (0.47–1.19)	0.24	0.78 (0.43–1.39)	0.4	0.68 (0.38–1.21)	0.2
≤65						
>65						
CEA (ng/mL)	1.21 (0.74–1.94)	0.44	1.24 (0.72–2.09)	0.42	1.39 (0.79–2.41)	0.24
≤5						
>5						
Number of lesions	1.19 (0.77–1.85)	0.44	0.92 (0.49–1.75)	0.8	0.98 (0.52–1.86)	0.94
≤4						
>4						
Diameter largest lesion (cm)	0.77 (0.49–1.2)	0.25	1.14 (0.63–2.1)	0.67	0.99 (0.56–1.78)	0.99
≤3						
>3						
Distribution of lesions						
Bilobar	1.19 (0.74–1.92)	0.48	1.03 (0.48–2.1)	0.99	0.84 (0.41–1.79)	0.64
Location of colon primary						
Right	1.53 (0.96–2.44)	0.07	0.72 (0.42–1.2)	0.22	0.79 (0.46–1.4)	0.39
Presentation of metastasis						
Synchronous	1.01 (0.57–1.77)	0.98	1.2 (0.44–3)	0.69	1.45 (0.52–3.6)	0.44
Perioperative chemotherapy						
Received	1.05 (0.33–3.37)	0.93	0.97 (0.22–3)	0.97	0.94 (0.21–2.92)	0.92
Never debulked group	Reference				Reference	
Partial debulked group	0.48 (0.27–0.85)	0.01			0.56 (0.29–1.03)	0.08
Subtotal debulked group	0.42 (0.23–0.77)	0.005			0.37 (0.17–0.8)	0.01
Combined partial + subtotal debulked group	0.46 (0.28–0.76)	0.002			0.48 (0.27–0.9)	0.02

HR, hazard ratio; BMI, body mass index; CEA, carcinoembryonic antigen. * Analysis was performed with the “never debulked” group as the reference group.

## Data Availability

The raw data supporting the conclusions of this article will be made available by the authors without undue reservation.

## Supplementary Materials

The following supporting information can be downloaded at: https://www.mdpi.com/article/10.3390/cancers16091730/s1, Table S1: Management of postoperative complications.
