# Supplementary material for: Debulking Hepatectomy for Colorectal Liver Metastasis Conveys Survival Benefit
Source: Cancers (Basel). 2024 Apr 29;16(9):1730. doi: 10.3390/cancers16091730 (PMC11083421; doi:10.3390/cancers16091730)
Supplement: Supplementary file 1 [file cancers-16-01730-s001.zip › cancers-2992822-supplementary.pdf]

Supplementary Table S1: Management of postoperative complications

Complication	Clavien-Dindo Classification	Management
Atrial fibrillation	II	Cardiology evaluation<br>Beta-blocker<br>Anticoagulation
ISGLS PHLF Grade A:<br>Elevation in bilirubin on POD 5	0	Observation
Infected biloma, abscess	IIIa	Antibiotics<br>IR drainage
Below knee DVT	II	Therapeutic anticoagulation
Ileus	I	Bowel rest<br>Electrolyte replacement
Prolonged Ileus	II	Bowel rest<br>Electrolyte replacement
Postoperative hypotension	II	Transient levophed infusion
ISGLS PHLF Grade B:<br>Development of ascites	0	Temporary diuretics
Atrial fibrillation	II	Cardiology evaluation<br>Beta-blocker<br>Anticoagulation
Candida, groin	II	Topical anti-fungal cream
PVT	II	Therapeutic anticoagulation
Hypovolemia, syncope	II	Fluid resuscitation<br>Telemetry
MCA stroke	IVb	ICU<br>Endotracheal intubation<br>Dialysis
PE		Therapeutic anticoagulation
Pelvic abscess	IIIa	Antibiotics<br>IR drainage
ISGLS PHLF Grade A:<br>Elevation in bilirubin on POD 5	0	Observation

ISGLS, international study group of liver surgery; PHLF, post-hepatectomy liver failure; DVT, deep vein thrombosis; PVT, portal vein thrombosis; MCA, middle cerebral artery; PE, pulmonary embolism; IR, interventional radiology; ICU, intensive care unit
